# Research on Aging: The International View From the Editors’ Desks

**DOI:** 10.1093/geroni/igab046.1391

**Published:** 2021-12-17

**Authors:** Kyungmin Kim, Jeffrey Burr, Changmin Peng

**Affiliations:** 1 Seoul National University, Seoul, Seoul-t'ukpyolsi, Republic of Korea; 2 McCormack Graduate School, Boston, Massachusetts, United States; 3 University of Massachusetts Boston, Boston, Massachusetts, United States

## Abstract

We review the scope, content, and focus of the peer-reviewed journal, Research on Aging (SAGE), publishing its 422nd volume this year. We will discuss how scholarship produced from researchers around the globe has changed over the years. Data on submissions, acceptance rates, and the important role of an international editorial board will be presented. The review process will be described, along with suggestions on how to increase chances of success when submitting original research. Although Research on Aging is sometimes considered to focus primarily on social gerontology, the scope in recent years has widened considerably, with manuscripts in aging studies published from such fields as economics, psychology, demography, public health, and public policy, as well as from sociology, and social work, among others. One of several special issues forthcoming in the journal will be described to demonstrate the possibilities for international impact.

